# Psychosocial impact at the time of a rare disease diagnosis

**DOI:** 10.1371/journal.pone.0288875

**Published:** 2023-07-28

**Authors:** Juan Benito-Lozano, Greta Arias-Merino, Mario Gómez-Martínez, Beatriz Arconada-López, Begoña Ruiz-García, Manuel Posada de la Paz, Verónica Alonso-Ferreira

**Affiliations:** 1 Institute of Rare Diseases Research (IIER), Instituto de Salud Carlos III (ISCIII), Madrid, Spain; 2 Universidad Nacional de Educación a Distancia (UNED), Madrid, Spain; 3 Spanish Federation of Rare Diseases (FEDER), Federación Española de Enfermedades Raras, Madrid, Spain; 4 The State Reference Center for Assistance to People Living with Rare Diseases and their Families (Creer), Centro de Referencia Estatal de Atención a Personas con Enfermedades Raras y sus Familias, dependiente del IMSERSO, Burgos, Spain; 5 Undiagnosed Diseases Network International (UDNI), Madrid, Spain; 6 Centro de Investigación Biomédica en Red de Enfermedades Raras (CIBERER), Madrid, España; University of A Coruña, SPAIN

## Abstract

Over half of all persons with rare diseases (RDs) in Spain experience diagnostic delay (DD) but little is known about its consequences. This study therefore aimed to analyze the psychological impact of obtaining a diagnosis of an RD, and to ascertain what social determinants are influenced and what the personal consequences are, according to whether or not patients experienced DD. Data were obtained from a purpose-designed form completed by persons registered at the Spanish Rare Diseases Patient Registry. The following were performed: a descriptive analysis; a principal component analysis (PCA); and logistic regressions. Results revealed that while searching for a diagnosis, people who experienced DD were more in need of psychological care than those diagnosed in less than one year (36.2% vs 23.2%; p = 0.002; n = 524). The PCA identified three principal components, i.e., psychological effects, social implications, and functional impact. Reducing DD would improve psychological effects, such as irritability (OR 3.6; 95%CI 1.5–8.5), frustration (OR 3.4; 95%CI 1.7–7.1) and concentration on everyday life (OR 3.3; 95%CI 1.4–7.7). The influence of the social implications and functional repercussions of the disease was greater in persons with DD (scores of 22.4 vs 20 and 10.6 vs 9.4, respectively) in terms of the difficulty in explaining symptoms to close friends and family (3.3 vs 2.9), and loss of independence (3.3 vs 2.9). In conclusion, this is the first study to analyze the psychosocial impact of diagnosis of RDs in Spain and one of few to assess it in the patients themselves, based on data drawn from a purpose-designed form from a national registry open to any RD. People affected by RDs who underwent DD experienced greater psychosocial impact than did those who were diagnosed within the space of one year.

## Introduction

Rare diseases (RDs) are defined by the European Union (EU) as any disease that has a prevalence of fewer than 5 cases per 10,000 population [1]. The different RDs may appear rare individually, but as a whole, they have an important impact on public healthcare services [2]. Moreover, for the people who suffer from them, RDs generally give rise to difficulties in performing daily tasks, problems with social and family relationships, and financial difficulties, among others [3–5].

The search for the diagnosis of an RD is a critical phase involving strong emotional stress [6], due to the pilgrimage from one clinical specialist to another, referrals to reference centers, and the perception of scant knowledge about RDs both among health professionals and in social and educational spheres [7, 8]. This so-called “diagnostic odyssey” is a long and tortuous process, during which people who live with RDs have to deal with the progression of their own disease, as well as delays in access to a possible treatment and/or early intervention program, among other problems [3, 4, 6, 9–13].

Among people who live with RDs, the absence of diagnosis and/or late or erroneous diagnosis generates feelings of frustration, anxiety, loneliness, fear and uncertainty, inability to make plans, loss of reproductive self-esteem due to poorly defined genetic risk, and guilt [4, 5, 8, 12, 14–16]. Frank has called it the “narrative of chaos” [17], characterized by a feeling that life will never improve and that one has no control over it [18]. In contrast, some of those affected by RDs develop greater tolerance to uncertainty, plus better coping and self-sufficiency skills, such as maintaining hope, re-creating positive images of the future, ignoring the severity of their ailment, searching for social support, and focusing on present possibilities [6, 9]. In the case of people that have spent many years awaiting a diagnosis, their interest in the prospect wanes with time, as they come to acknowledge that their situation may not change substantially [18, 19].

Obtaining a diagnosis has a strong emotional impact on patients [13, 14]. Sometimes, diagnosis will lead to a change in the treatment being administered and referral to specialized clinical care, all of which results in an improvement in the quality of life of RD-sufferers and their process of acceptance [15, 20]. It is at this stage when these people demand to have basic, easily understandable information about the disease itself, their future, and any possible available treatments [16, 21, 22]. There is, however, a dearth of such information, which may lead to patients feeling helpless and turning to untrustworthy information in some sources of the Internet [23], where the quality of information about RD has not been fully validated [24]. Conversely, there may also be positive source of credible information like websites sponsored by government agencies (e.g. Orphanet and GARD, among others). In addition, they seek to establish new relationships with persons or groups that share the same disease and make contact with established support networks [6, 8, 9, 20, 22, 25]. Access to support networks and patient associations is easier if there is a diagnosis and is an important element of emotional support, though approximately only half of RDs have patient associations [19, 26, 27]. Unfortunately, most people with RDs do not receive the support of professional psychologists [9, 23].

In Spain, over half of all people with RDs experience diagnostic delay (DD), and the mean time spent in obtaining a diagnosis is in excess of 5 or even 6 years [28, 29]. Some of the main determinants of this DD were identified: having to travel to see a specialist other than that usually consulted in the patient’s home region; visiting more than 10 specialists; being diagnosed in a different region or suffering from a RD of the nervous system [30]. However there continues to be scant knowledge about the consequences that this diagnostic odyssey and the obtaining of the diagnosis have on persons, especially at a psychosocial level [9, 31], i.e., how they have been affected, both emotionally and with respect to their immediate environment. Although some studies have been carried out at an international level, these tend to focus on specific RDs rather than address RDs as a whole [32, 33].

Hence, the aim of this study was: first, to analyze the psychological impact of obtaining the diagnosis of an RD and how this impact influences the person affected, depending on whether or not he/she has experienced DD; and second, to ascertain how such a diagnosis affects the social aspects of life and what personal consequences it has for the people diagnosed with an RD.

## Methods

### Study design, setting and procedure

We compared the data of people with DD vs those who did not have DD. Those who suffered DD were defined as anyone with an RD who was waiting more than one year between the date of the first medical visit about his/her symptoms and the date on which diagnosis was obtained [3]: for people without DD, this period was set at one year or less. This observational study analysed retrospective data. A specific form covering DD and psychosocial impact was purpose-designed by the authors and host at the Spanish Rare Diseases Patient Registry of the Carlos III Institute of Health (*Instituto de Salud Carlos III/ISCIII*), a nationwide registry open to any RD. The form contained questions covering family and marital environment, psychological and health care, psychological aspects, and personal and social determinants. The form was a self-reported electronic survey in Spanish.

The inclusion criteria were people i) diagnosed with an RD; ii) who signed an informed consent form; iii) who were entered on the Spanish Rare Diseases Patient Registry as at 1 January 2022; iv) who voluntarily agreed to participate in the study; and v) resident in Spain [30]. The invitation to participate was sent by email to all people enrolled in the Spanish Rare Diseases Patient Registry (personal data included in the process of enroll in the Registry). All participants were over 18 years old. No incentives were given according to the guidance of the Ethics Committee.

The Spanish Rare Diseases Patient Registry and the present study were approved by the Committee for Ethical Research of the Institute of Health Carlos III (CEI PI74_2016 and CEI PI 58_2021-v2). There are two ways to enrol in the Registry: i) patients themselves with a confirmed diagnosis of a RD and ii) professionals participating in research networks and medical societies that have an agreement with the ISCIII. All Registry patients have a written informed consent.

### Information sought

The study variables were divided into 5 blocks:

- the person: sex, age, type of RD (classified by organ or main system affected, as per International Classification of Diseases 10th Revision criterion), and dates of first medical visit and diagnosis;
- family and marital environment: social isolation, care by the family, family relationships, and couple’s relationship (got worse, stayed the same, got better);
- psychological and health care: need to see a psychologist prior to diagnosis, having been seen by a psychologist (Yes/No), need to see a psychologist after diagnosis, and health status in the 12 months after diagnosis (got worse, stayed the same, got better);
- psychological aspects: depressed mood, frustration, anxiety, fear, uncertainty about the future, irritability, indecision, difficulty falling asleep, muscle tension, concentration on everyday life, and changes in eating habits (got worse, stayed the same, got better); and,
- disease-related personal and social determinants that influenced how the patient felt: difficulty in explaining symptoms to close friends and family, difficulty in justifying absences (occupational or educational) for medical reasons, isolation due to the difficulty of finding other people with the same diagnosis, lack of psychological support, difficulty in everyday planning, difficulty in accessing aid, financial difficulties due to the disease, loss of independence, loss of abilities and loss of opportunities (occupational or educational).

Replies were scored on a 5-point ordinal scale ranging from 1 (not at all) to 5 (very much).

We used a computer-assisted web interviewing method [34] and questionnaires adapted to participants with visual impairment. A health psychologist used a telephone survey to interview participants who had difficulties in coping with new technologies.

### Sample

Forms with more than four unanswered questions from blocks IV and V were discarded. Participants were contacted by telephone and e-mail in order to obtain the missing information. If the questions from blocks IV and V were still incomplete after the contact those forms were finally discarded (median of missing answers = 3; IQR 1–7). These questions are not compulsory, and being of a sensitive nature may elicit anxiety and thus have a high non-response rate [35]. We performed a quality analysis, comparing the initial sample against the sample with blocks IV and V fully completed, and found no noteworthy differences between the two (S1 Table). Lastly, we analyzed the latter sample with the higher level of completeness. The complete list of included RD of the sample can be found in S2 Table.

### Data-analysis

A descriptive analysis was performed, using the Chi-squared and Mann-Whitney U tests to assess differences between persons with and without DD. Odds ratios (ORs) were calculated with each of the variables using logistic regressions. Principal component analysis (PCA) was applied to the block-IV and -V variables, to prevent excessive correlation in the replies. The aim was to reduce these to a minimum number of variables that would explain the maximum possible variability of the data. Eigenvalues ≥1.0 and factor loading coefficients ≥ 0.4 were considered. Varimax orthogonal rotation with Kaiser normalization was used. In the case of the components created with the block-V variables, we calculated a component score that was the sum of the replies for each of the variables (1–5). All analyses were performed using the IBM SPSS v28 computer software package and were replicated in Stata 15.

## Results

A total of 805 persons diagnosed with RDs participated in the study. The final sample comprised 524 people with RDs. Its characteristics are shown in Table 1 (age $\bar{x}$ = 50.5; 61.6% women; 343 people with DD and 181 diagnosed within a year).

**Table 1:** Characteristics of the sample of 524 people affected by RDs in Spain (variables of block I).

		With diagnostic delay % (n)	Diagnosed within a year % (n)	p value (Chi-squared test)	OR (95%CI)
**Sex**	Men**	36.7 (126)	41.4 (75)	0.293	
	Women	63.3 (217)	58.6 (106)		1.2 (0.8–1.8)
**Type of RD**	Diseases of the musculoskeletal system and connective tissue**	9 (31)	16 (29)	0.002*	
	Diseases of the nervous system	31.8 (109)	25.4 (46)		2.2 (1.2–4.1)
	Congenital malformations, deformations, and chromosomal abnormalities	14.0 (48)	7.2 (13)		3.5 (1.5–7.9)
	Diseases of the eye and adnexa	18.4 (63)	17.7 (32)		1.8 (0.9–3.6)
	Endocrine, nutritional, and metabolic diseases	10.5 (36)	7.2 (13)		2.6 (1.1–6.0)
	Others	16.3 (56)	26.5 (48)		1.1 (0.6–2.1)
**Age at diagnosis (years)**	<15**	5.0 (17)	11.0 (20)	0.001*	
	15–29	19.0 (65)	28.7 (52)		1.5 (0.7–3.1)
	30–44	42.6 (146)	36.5 (66)		2.6 (1.3–5.4)
	>45	33.5 (115)	23.8 (43)		3.1 (1.5–6.7)

RD: rare disease

*An asterisk indicates significance at p<0.05

** Reference group

Table 2 shows that during the diagnostic process, a higher percentage of persons with DD needed psychological care than did persons diagnosed within a year (36.2% vs 23.2%). Even so, obtaining a diagnosis in those persons who experienced DD, was associated with an improvement in their feelings of frustration (16.6% of persons who suffered DD improved vs 6.1% of persons diagnosed within a year), fear (15.5% vs 7.2%), social isolation (14.4% vs 8.4%), difficulty of concentrating on everyday life (12.2% vs 3.9%), and irritability (12% vs 3.9%).

**Table 2:** Psychological impact of diagnosis on people affected by RDs in Spain.

		With diagnostic delay % (n)	Diagnosed within a year % (n)		p value (Chi-squared test)	OR (95%CI)
**SOCIAL AND FAMILY ENVIRONMENT (BLOCK II)**						
Social isolation (n = 512)	Got worse	26.7 (89)	36.3 (65)		0.033*	0.7 (0.4–1.0)
	Stayed the same**	55.3 (184)	49.7 (89)			
	Got better	14.4 (48)	8.4 (15)			1.5 (0.8–2.9)
	Not applicable	3.6 (12)	5.6 (10)			
Care provided by family (n = 511)	Got worse	7.7 (26)	6.9 (12)		0.893	1.2 (0.6–2.5)
	Stayed the same**	56 (188)	59.4 (104)			
	Got better	34.2 (115)	32 (56)			1.1 (0.8–1.7)
	Not applicable	2.1 (7)	1.7 (3)			
Family relationships (n = 519)	Got worse	11.7 (40)	12.9 (23)		0.652	0.9 (0.5–1.5)
	Stayed the same**	64.8 (221)	62.9 (112)			
	Got better	21.7 (74)	23.6 (42)			0.9 (0.6–1.4)
	Not applicable	1.8 (6)	0.6 (1)			
Couple’s relationship (n = 500)	Got worse	17.2 (56)	18.4 (32)		0.329	1.0 (0.6–1.7)
	Stayed the same**	47.5 (155)	51.7 (90)			
	Got better	17.5 (57)	18.4 (32)			1.0 (0.6–1.7)
	Not applicable	17.8 (58)	11.5 (20)			
**PSYCHOLOGICAL CARE AND HEALTH STATUS (BLOCK III)**						
Need for psychological care before diagnosis (n = 524)	No	63.8 (219)	76.8 (139)		0.002*	
	Yes	36.2 (124)	23.2 (42)			1.9 (1.2–2.8)
Need for psychological care after diagnosis (n = 524)	Has decreased	11.1 (38))	7.7 (14)		0.225	1.4 (0.7–2.6)
	Stayed the same**	55.7 (191	52.5 (95))			
	Has increased	33.2 (114)	39.8 (72)			0.8 (0.5–1.2)
Receiving psychological care (n = 524)	No**	45.5 (156)	53.6 (97)		0.077	
	Yes	54.5 (187)	46.4 (84)			1.4 (1.0–2.0)
Health status within 12 months after diagnosis (n = 524)	Got worse	27.7 (95)	35.4 (64)		0.011*	0.6 (0.4–0.9)
	Stayed the same**	51.9 (178)	38.1 (69)			
	Got better	20.4 (70)	26.5 (48)			0.6 (0.4–0.9)
**PSYCHOLOGICAL ASPECTS (BLOCK IV)**						
Depressed mood (n = 524)	Got worse	46.9 (161)	54.1 (98)		0.227	0.8 (0.5–1.2)
	Stayed the same**	39.4 (135)	35.9 (65)			
	Got better	13.7 (47)	9.9 (18)			1.3 (0.7–2.3)
Frustration (n = 524)	Got worse	50.1 (172)	51.9 (94)		0.002*	1.2 (0.8–1.8)
	Stayed the same**	33.2 (114)	42 (76)			
	Got better	16.6 (57)	6.1 (11)			3.4 (1.7–7.1)
Anxiety (n = 524)	Got worse	45.8 (157)	53.6 (97)		0.056	0.8 (0.6–1.2)
	Stayed the same**	38.5 (132)	37.6 (68)			
	Got better	15.7 (54)	8.8 (16)			1.7 (0.9–3.3)
Fear (n = 524)	Got worse	44 (151)	51.9 (94)		0.018*	0.9 (0.6–1.3)
	Stayed the same**	40.5 (139)	40.9 (74)			
	Got better	15.5 (53)	7.2 (13)			2.2 (1.1–4.3)
Uncertainty about the future (n = 524)	Got worse	64.4 (221)	68.5 (124)		0.312	0.9 (0.6–1.4)
	Stayed the same**	25.7 (88)	25.4 (46)			
	Got better	9.9 (34)	6.1 (11)			1.6 (0.7–3.5)
Irritability (n = 524)	Got worse	40.8 (140)	42.5 (77)		0.007*	1.1 (0.7–1.6)
	Stayed the same**	46.9 (161)	53.6 (97)			
	Got better	12.2 (42)	3.9 (7)			3.6 (1.5–8.5)
Concentration on everyday life (n = 524)	Got worse	40.2 (138)	47 (85)		0.006*	0.9 (0.6–1.3)
	Stayed the same**	47.5 (163)	49.2 (89)			
	Got better	12.2 (42)	3.9 (7)			3.3 (1.4–7.7)
Difficulty in making decisions (n = 524)	Got worse	28.3 (97)	34.3 (62)		0.055	0.8 (0.6–1.3)
	Stayed the same**	55.1 (189)	56.4 (102)			
	Got better	16.6 (57)	9.4 (17)			1.8 (1.0–3.3)
Difficulty falling sleep (n = 524)	Got worse	41.4 (142)	44.2 (80)		0.672	0.9 (0.6–1.3)
	Stayed the same**	51.3 (176)	50.3 (91)			
	Got better	7.3 (25)	5.5 (10)			1.3 (0.6–2.8)
Muscle tension (n = 524)	Got worse	44.3 (152)	49.7 (90)		0.307	0.8 (0.6–1.2)
	Stayed the same**	47.8 (164)	45.3 (82)			
	Got better	7.9 (27)	5 (9)			1.5 (0.7–3.3)
Changes in eating habits (decrease or increase in appetite) (n = 524)	Got worse	32.9 (113)	37 (67)		0.224	0.9 (0.6–1.3)
	Stayed the same**	58.9 (202)	58.6 (106)			
	Got better	8.2 (28)	4.4 (8)			1.8 (0.8–4.2)

Not applicable means that the information requested does not apply to the person who is filling out the form.

RD: rare disease

* An asterisk indicates significance at p<0.05

** Reference group

In terms of the maximum score for determinants in block V (what influenced them the most), people with DD outweighed those without DD in the following: difficulty in explaining symptoms to close friends and family (28% vs 17.7%); difficulty in justifying absences (occupational or educational) for medical reasons (33.9% vs 23.8%); loss of opportunities (occupational or educational) (42.6% vs 33.1%); and lack of psychological support (29.7% vs 21%) (Table 3).

**Table 3:** RD-related social and personal determinants (variables of block V; n = 524).

		With diagnostic delay % (n)	Diagnosed within a year % (n)	OR (95%CI)
Difficulty in explaining the symptoms to close friends and family	1-Not at all	17.5 (60)	28.2 (51)	1.23 (1.09–1.39)
	2	9.3 (32)	12.2 (22)	
	3	23 (79)	18.8 (34)	
	4	22.2 (76)	23.2 (42)	
	5-Very much	28 (96)	17.7 (32)	
Difficulty in justifying absences from work or school	1-Not at all	28.6 (98)	38.7 (70)	1.17 (1.05–1.31)
	2	7 (24)	10.5 (19)	
	3	13.7 (47)	9.4 (17)	
	4	16.9 (58)	17.7 (32)	
	5-Very much	33.9 (116)	23.8 (43)	
Isolation due to the difficulty of finding other people with the same diagnosis	1-Not at all	23.9 (82)	31.5 (57)	1.16 (1.04–1.31)
	2	8.7 (30)	13.3 (24)	
	3	19.8 (68)	17.1 (31)	
	4	16.6 (57)	14.9 (27)	
	5-Very much	30.9 (106)	23.2 (42)	
Lack of psychological care	1-Not at all	23.9 (82)	33.7 (61)	1.21 (1.07–1.36)
	2	9 (31)	13.8 (25)	
	3	20.4 (70)	17.7 (32)	
	4	16.9 (58)	13.8 (25)	
	5-Very much	29.7 (102)	21 (38)	
Difficulty in planning everyday life	1-Not at all	24.5 (84)	29.8 (54)	1.08 (0.96–1.21)
	2	13.4 (46)	11.6 (21)	
	3	16.6 (57)	19.9 (36)	
	4	20.1 (69)	15.5 (28)	
	5-Very much	25.4 (87)	23.2 (42)	
Difficulty accessing resources or benefits	1-Not at all	20.1 (69)	22.1 (40)	1.07 (0.95–1.20)
	2	7.9 (27)	11.6 (21)	
	3	16 (55)	15.5 (28)	
	4	16.9 (58)	15.5 (28)	
	5-Very much	39.1 (134)	35.4 (64)	
Financial difficulties due to the disease	1-Not at all	30.9 (106)	38.7 (70)	1.14 (1.02–1.28)
	2	13.4 (46)	13.8 (25)	
	3	14.9 (51)	15.5 (28)	
	4	13.4 (46)	12.7 (23)	
	5-Very much	27.4 (94)	19.3 (35)	
Loss of independence due to the disease	1-Not at all	19.8 (68)	30.9 (56)	1.17 (1.04–1.32)
	2	10.2 (35)	11.6 (21)	
	3	18.7 (64)	16.6 (30)	
	4	18.7 (64)	12.7 (23)	
	5-Very much	32.7 (112)	28.2 (51)	
Loss of abilities (limitations) due to the disease	1-Not at all	7.6 (26)	17.1 (31)	1.19 (1.04–1.36)
	2	9.6 (33)	9.4 (17)	
	3	21.3 (73)	19.3 (35)	
	4	23 (79)	21 (38)	
	5-Very much	38.5 (132)	33.1 (60)	
Loss of work or academic opportunities due to the disease	1-Not at all	22.7 (78)	33.1 (60)	1.17 (1.05–1.30)
	2	9 (31)	10.5 (19)	
	3	11.4 (39)	11.6 (21)	
	4	14.3 (49)	11.6 (21)	
	5-Very much	42.6 (146)	33.1 (60)	

RD: rare disease

**Fig 1:**
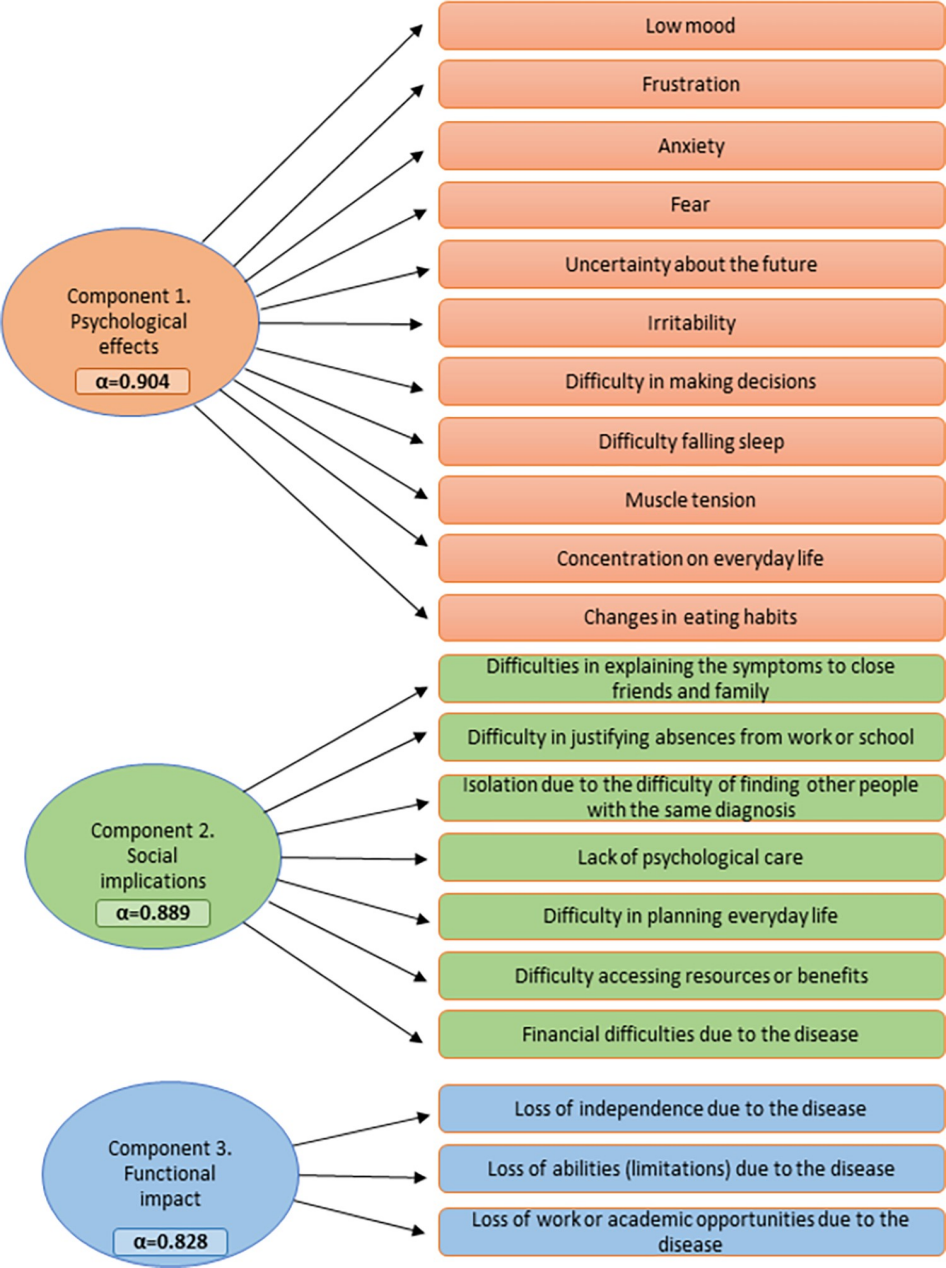
Principal component analysis (PCA) of psychological impact of diagnostic delay on people affected by RDs.

PCA identified three principal components that accounted for 60.4% of total variance. Component 1 was labeled as psychological effects, component 2 as social implications, and component 3 as functional repercussions of the disease. The components displayed good internal consistency (Cronbach’s alpha>0.8) (Fig 1).

In component 1 (psychological effects), persons who experienced DD registered higher ORs in “got better” compared to “stayed the same” in the following variables: irritability (OR 3.6; 95%CI 1.5–8.5); frustration (OR 3.4; 95%CI 1.7–7.1); concentration on everyday life (OR 3.3; 95%CI 1.4–7.7); fear (OR 2.2; 95%CI 1.1–4.3); and difficulty in making decisions (OR 1.8; 95%CI 1.0–3.3) (Table 2).

**Fig 2:**
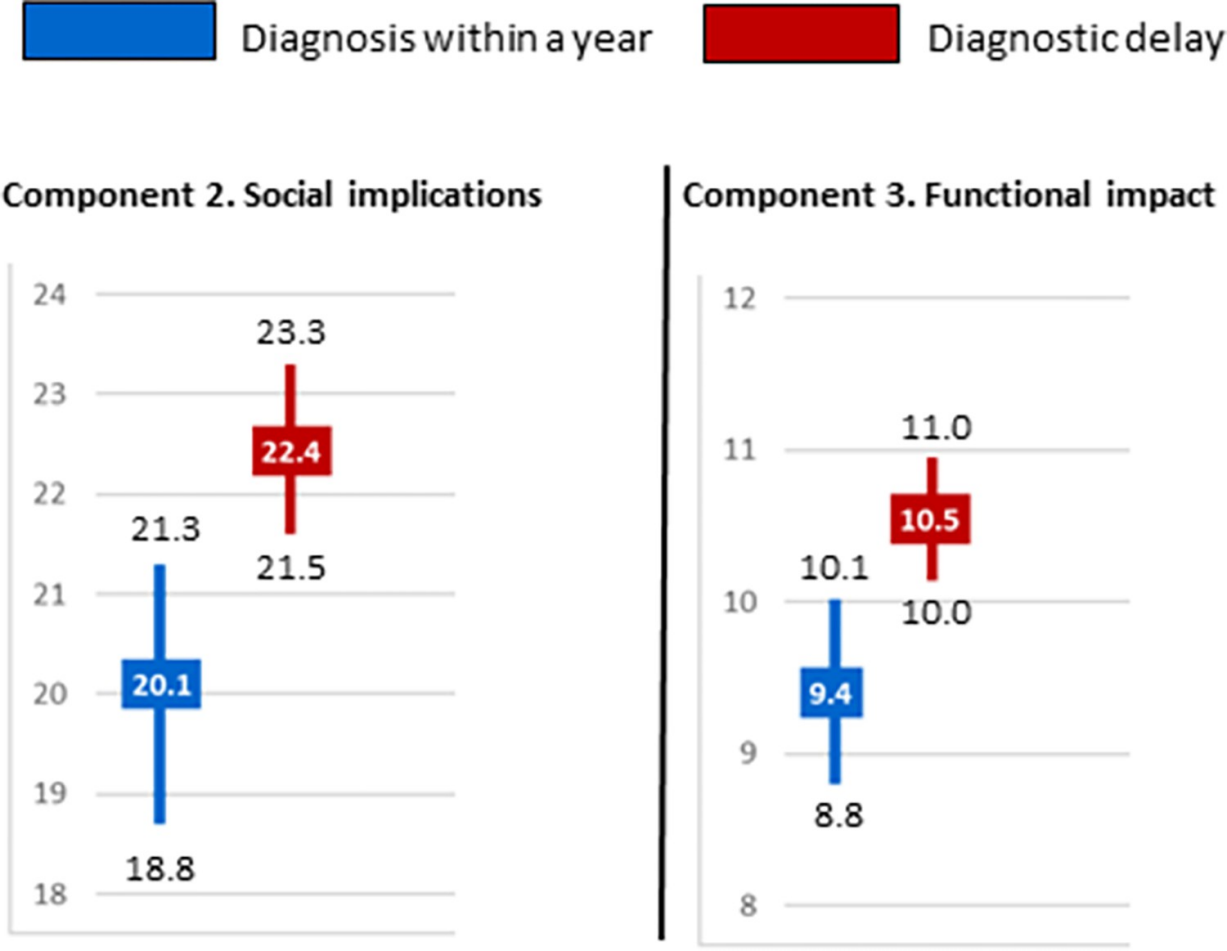
Social implications (sum of component 2) and functional impact (sum of component 3) on persons with diagnostic delay versus those diagnosed within a year. Mean is represented in squares and CI in bars.

**Fig 3:**
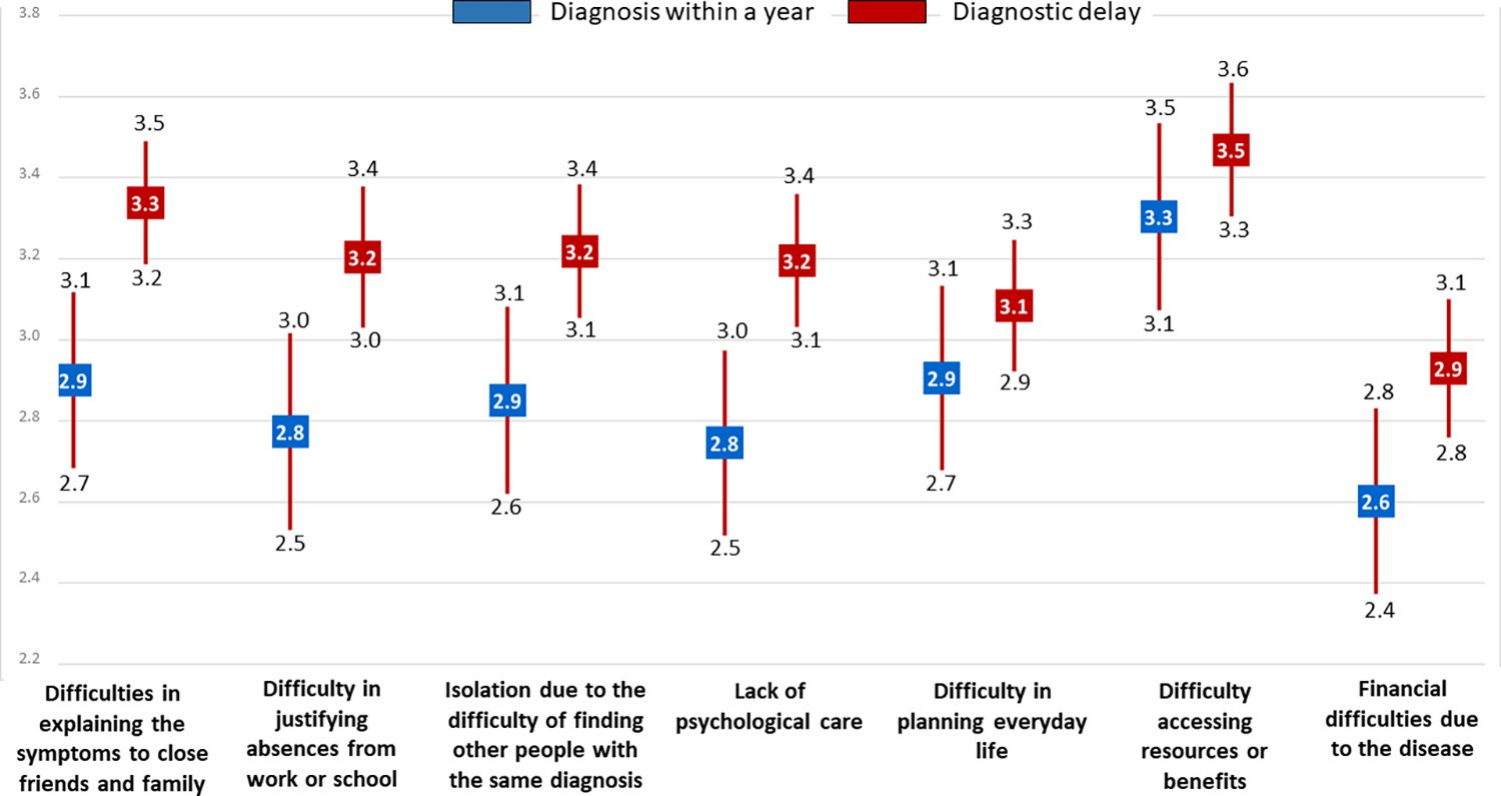
Social implications variables (component 2) on persons with diagnostic delay versus those diagnosed within a year. Mean is represented in squares and CI in bars.

The mean total score for component 2 (social implications) was significantly higher in people with DD than diagnosed within a year, indicating that these variables exerted a greater influence on how the former felt (22.4 vs 20.1) (Fig 2), as was likewise observed for component 1. Similarly, women were influenced to a greater extent, regardless of whether they had experienced DD (median 25 vs 21) or been diagnosed in less than a year (22 vs 16). In addition, people with DD reported a higher median than did those without DD in cases of endocrine disorders (26.5 vs 19) or congenital malformation (28.5 vs 18.0) (Table 4). Among persons who experienced DD, the influence of component-2 variables was greater in the following areas: difficulty in explaining the symptoms to close friends and family (3.3 vs 2.9); difficulty in justifying absences (occupational or educational) for medical reasons (3.2 vs 2.8); and lack of psychological support (3.2 vs 2.8) (Fig 3).

**Table 4:** Influence of sex and type of RD on social implications (component 2) and functional impact (component 3; n = 524).

				n	Median	IQR	p value (Mann-Whitney U test)
**Social implications (Component 2)**	Sex	Diagnostic delay	Men	126	21	12–29	0.004*
			Women	217	25	18–30	
		Diagnosis within a year	Men	75	16	9–26	0.003*
			Women	106	22	15–28	
	Type of RD	Diseases of the musculoskeletal system and connective tissue	DD	31	27	20–30	0.317
			<1 year	29	23	18–29	
		Diseases of the nervous system	DD	109	24	18–30	0.091
			<1 year	46	21.5	13–27	
		Congenital malformations, deformations and chromosomal abnormalities	DD	48	28.5	22–32	0.012*
			<1 year	13	18	7–27	
		Diseases of the eye and adnexa	DD	63	14	9–21	0.556
			<1 year	32	13.5	9–19.5	
		Endocrine, nutritional and metabolic diseases	DD	36	26.5	20–31	0.005*
			<1 year	13	19	14–24	
		Others	DD	56	22	16–29	0.969
			<1 year	48	25	12.5–29	
**Functional impact (Component 3)**	Sex	Diagnostic delay	Men	126	11	8–14	0.198
			Women	217	12	8–15	
		Diagnosis within a year	Men	75	9	5–11	0.06*
			Women	106	10.5	6–14	
	Type of RD	Diseases of the musculoskeletal system and connective tissue	DD	31	11	8–13	0.87
			<1 year	29	10	9–14	
		Diseases of the nervous system	DD	109	12	9–15	0.033*
			<1 year	46	11	6–13	
		Congenital malformations, deformations and chromosomal abnormalities	DD	48	12	8–14	0.060*
			<1 year	13	9	3–12	
		Diseases of the eye and adnexa	DD	63	10	7–13	0.566
			<1 year	32	10	6–12.5	
		Endocrine, nutritional and metabolic diseases	DD	36	12.0	8.5–14.5	<0.001*
			<1 year	13	5.0	4–9	
		Others	DD	56	9.0	6–13	0.743
			<1 year	48	9.0	6–14.5	

RD = rare disease; DD = diagnostic delay; <1 year = diagnosis within a year; IQR = interquartile range

*An asterisk indicates significance at p0<0.05

**Fig 4:**
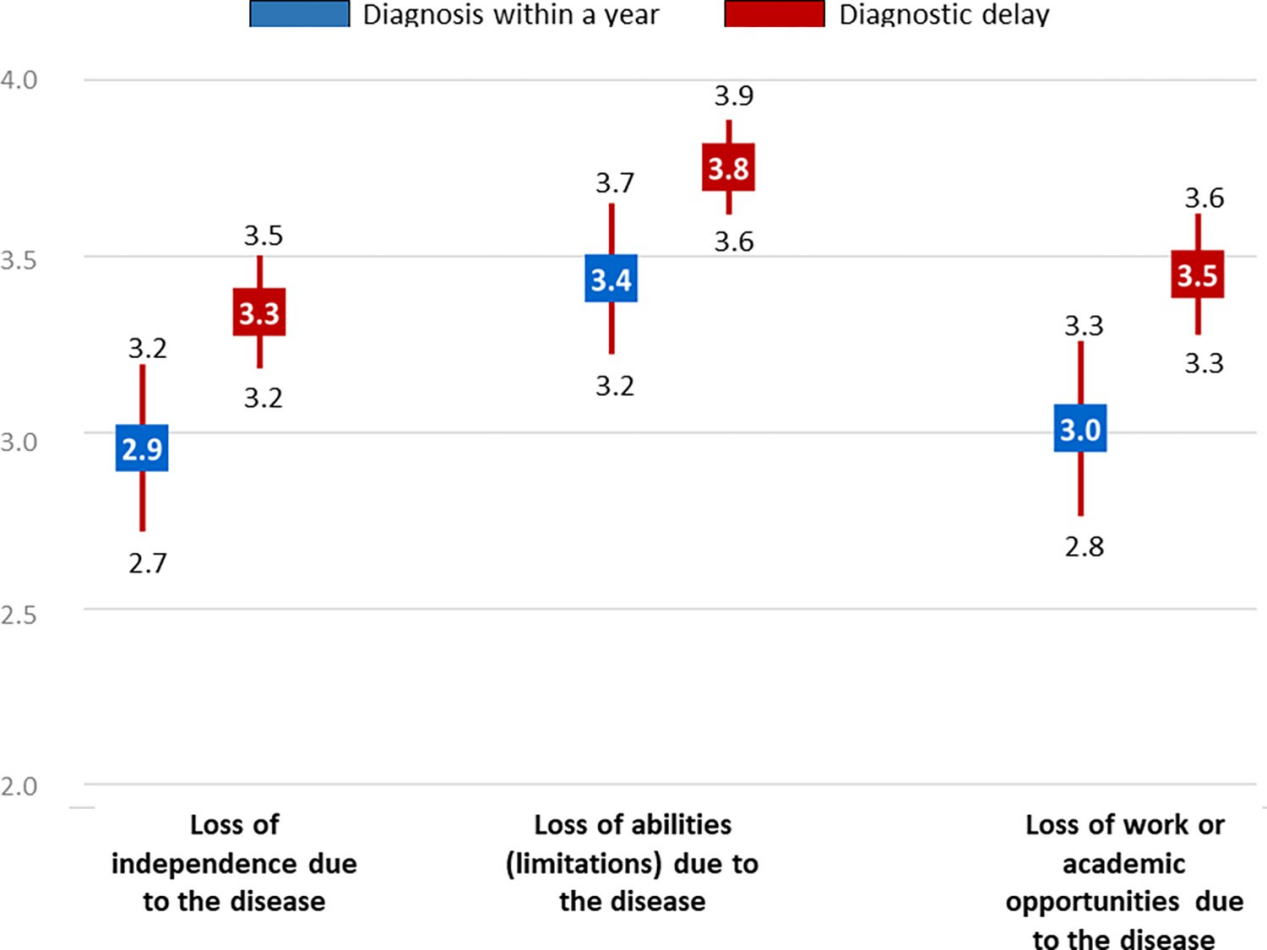
Functional repercussions of the disease variables (component 3) on persons with diagnostic delay versus those diagnosed within a year. Mean is represented in squares and CI in bars.

The mean score for functional repercussions due to the disease (component 3) was, as in the case of social implications (component 2), significantly higher in people with than in those without DD (10.5 vs 9.4) (Fig 2). In addition, the impact was greater when the person suffered from a disorder of the nervous system (12 with delay vs 11 without delay), endocrine disease (12 vs 5) or a congenital malformation (12 vs 9) (Table 4). Among persons with DD, the variable-by-variable analysis showed the influence to be greater in loss of independence due to the disease (3.3 vs 2.9), and loss of opportunities (occupational or educational) (3.5 vs 3.0) (Fig 4).

## Discussion

This is one of very few studies which examine the psychosocial impact of being informed of the diagnosis of an RD from the stance of the person affected, rather than that of his/her parents, family members or informants (another relative, tutor, caregiver, etc.). Furthermore, this impact was analyzed according to whether the patient in question had or had not experienced a delay in obtaining his/her diagnosis. As it is a study in which the patients answer about themselves, is usual to find more people who received the diagnosis in the young-adult stage (30–44 years).

As mentioned above, the psychosocial impact is generally greater among persons with DD, thus highlighting the importance of reducing the time taken until an RD is diagnosed. During the search for their diagnosis, people who experienced a delay needed psychological care to a greater extent than did those who were diagnosed within the space of a year. Notwithstanding this, the fact of having a diagnosis meant that persons with DD had a greater likelihood of improving in psychological variables such as irritability, frustration, concentration on everyday life, fear and difficulty in making decisions. Moreover, persons who experienced delay in the diagnosis of their RDs reported greater difficulties in their social context, such as: difficulty in explaining symptoms to close friends and family; difficulty in justifying absences (occupational or educational) for medical reasons; and lack of psychological support. Similarly, the effect of functional repercussions due to the disease was also greater in persons who took longer to be diagnosed. What affected them most, was the loss of independence due to the advance of their disease, and the loss of occupational or educational opportunities. Perceived health status remained unaltered to a greater degree in cases where DD had been experienced.

While seeking a diagnosis, people who experienced delay needed more psychological care, something that was clearly observed by this and other previous studies [28, 36, 37]. During this search period, people with RDs may receive erroneous diagnoses and treatments, in what has been considered a diagnostic odyssey [22]. Some of the causes of this DD are the lack of scientific knowledge surrounding RDs as a whole, the heterogeneity of RD or the unavailability of ad hoc diagnostic tests, among others [9, 30]. While, during all this time they watch how their condition deteriorate as they wait for an answer [4] and this may in turn have severe repercussions on their mental health, yet despite this, it is estimated that only one third of persons with RDs receive the psychological support they need [33, 37]. In this context, the public provision of professional psychological care to RD patients is clearly inadequate [33]. Spain has the lowest ratio in the EU, with 5.58 clinical psychologists per 100,000 population [38], and one third of patients reported that the country’s National Health System did not cover this need [32].

However, once the diagnosis is known, the possibilities of improvement in aspects of a psychological nature are higher among people who take over a year to obtain their diagnosis. Specifically, these psychological aspects are irritability, frustration, concentration on everyday life, fear, and difficulty in making decisions. The time of diagnosis marks a turning point in the life of persons with RDs. On the one hand, some persons experience feelings of hope, relief, validation, legitimation, and empowerment [31]. When it involves a disease whose natural history is known, these people feel relieved at finally having the possibility of being better understood, being able to better manage their life, adapting to a new normality, and taking pragmatic decisions based on their prognosis [5, 6, 8, 15, 31, 39]. People with a diagnosis become socially visible and recognized [4]. In contrast, diagnosis may have a negative impact on their emotional state because they now face the loss of hope of a possible recovery and/or the lack of treatment, and it generates feelings of depression, anxiety, blame, denial, discrimination, and financial stress, among other things [6].

The influence of the social implications of the disease is greater among persons who experience a delay in the diagnosis of their RDs, affecting all the aspects consulted but the following in particular: difficulty in explaining symptoms to close friends and family; difficulty in justifying absences (occupational or educational) for medical reasons; and lack of psychological support. The diagnosis of an RD can involve adverse effects, such as changes in the person’s social network [31]. Moreover, the lack of knowledge about an RD may generate a barrier effect in social aspects, which could have an impact on discrimination and participation in daily life [40]. In this respect, the overall impact of social implications is greater in women than in men: this finding is in line with previously published results which indicate gender inequality [8, 28, 32].

RDs frequently pose a challenge when it comes to tackling daily activities. The functional repercussions of the disease were greater in persons who experienced DD, especially in terms of loss of independence due to the disease and loss of occupational or educational opportunities. This disruption in their daily routine is pointed out in other studies as one of the greatest impacts on the emotional and social sphere [41]. Among the possible causes would presumably be the worsening of the disease or its symptoms, and the fact of not receiving treatments, or if these were being received, of their being inappropriate [28]. This has a direct influence on the loss of personal autonomy, and on emotional wellbeing. Added to this is the incomprehension of the patient’s immediate circle, a lack of credibility or ignorance [42], and usually leaves him/her unable to find an appropriate response in the existing public services [32]. In the workplace, there tends to be a lack of correlation between the person’s ability and training, and the job obtained [43], while in the educational sphere, the lack of access to material and human resources and failure to adapt to pupils’ needs are the most noteworthy causes of the loss of opportunities [28, 43, 44].

RDs, for the most part chronic and complex, have a generalized impact on the people that suffer from them, on public health systems, and on society as a whole. Given the paucity of clinical knowledge about these diseases, immediately after diagnosis is made, people and their families demand to have basic and comprehensible information about expectations and future health-related steps [21]. Health systems therefore need to be improved, since people who are undergoing a diagnostic odyssey or have been recently diagnosed, have a series of unmet psychological, social, personal, health, information, and care-related needs [45]. For example, more specialized training in RD for clinicians, more mental health services for people with RD, or putting people in contact with associations for patients with undiagnosed diseases. Indeed, the psychosocial impact of the diagnosis is high in people with RDs, and the difficulties that they face in their daily lives have many points in common, despite the differences that divide them as a group.

Among this study’s limitations is a possible recall bias. Due to the fact that the answers were retrospective, some of the interviewees might possibly have been unable to accurately remember their physical state and psychological and social conditions, essentially in the pre-diagnosis stage or when diagnosis was made many years ago. Other studies have been able to identify biases in replies about earlier stages in life, depending on the interviewee’s current stage in life, e.g., feelings of desperation, drama, worry, fear, and depression ascribed to the period prior to diagnosis by people who are currently reporting more negative perceptions of health, and vice-versa [5]. Accordingly, this could have influenced them, leading them to form a more negative perception of their past situation. Other possible limitations are: on the one hand, the lack of representation of certain RDs, since the study addressed all RDs as a whole; and on the other, the heterogeneity of the RDs that were included.

This study’s main strength lies in analyzing the psychosocial impact at the time of diagnosis of the people with RDs, since very few studies have approached the topic from this angle. Although there are other studies that analyze this same topic from the perspective of parents, family members or caregivers, it is nevertheless important to show the diagnostic odyssey from patients’ standpoint [3], in order to ascertain the factors that affect them and the real impact of diagnosis [29]. It should also be stressed here that, to encourage participation in the study and mitigate possible selection bias, forms adapted to the visually impaired were circulated, and an expert psychologist administered a telephone survey to anyone who requested this because of difficulties experienced in coping with new technologies. Lastly, in the Spanish context, this is the first study to analyze the psychological impact on people with RD, using a nationwide registry, open to all RDs, with confirmed diagnosis in clinical reports, as its data-source. Gaining insight into the psychosocial impact in the diagnostic setting of people with RDs is crucial when it comes to enhancing the intervention and services offered to such patients.

## Conclusions

This is the first study to analyze the psychosocial impact at the time of the diagnosis of people affected by RDs in Spain and registered in a national patient registry, using a purpose-designed form which directly recorded their experience, and one of few to assess it in the patients themselves. In general, psychological impact was high in persons with RDs, especially in those who took more than a year to obtain their diagnosis. In this respect, reducing the DD would improve psychological aspects, such as irritability or frustration. Persons who experienced DD, not only displayed a greater need for psychological care during the time spent searching for a diagnosis, but once this had been obtained, also felt the social implications (e.g., difficulty in explaining symptoms to close friends and family, or justifying absences for medical reasons) and functional repercussions of the disease (e.g., loss of independence and loss of occupational or educational opportunities) more acutely. More in-depth study is needed into the impact of DD on the life of persons with RDs and their families, in other settings, such as occupational, educational and financial.

## Supporting information

S1 Table Quality analysis comparing the sample with block-IV and-V questions completed (n = 524) to that with these questions uncompleted (n = 281). (DOCX)

S2 Table Table showing the complete list of included RD in the study. (DOCX)
