# Supplementary material for: Psychosocial impact at the time of a rare disease diagnosis
Source: PLoS One. 2023 Jul 28;18(7):e0288875. doi: 10.1371/journal.pone.0288875 (PMC10381039; doi:10.1371/journal.pone.0288875)
Supplement: S1 Table — (DOCX) [file pone.0288875.s001.docx]

**Supporting information**

**S1 Table**: Quality analysis comparing the sample with block-IV and-V questions completed (n=524) to that with these questions uncompleted (n=281)

|  |  | **Sample 281 n (%)** | **Sample 524 n (%)** | **p value (Chi-squared test)** |
| --- | --- | --- | --- | --- |
| **Delay** | Diagnosis within 1 year | 100 (35.6) | 181 (34.5) | 0.767 |
|  | Diagnostic delay | 181 (64.4) | 343 (65.4) |  |
| **Sex** | Men | 105 (37.3) | 201 (38.3) | 0.782 |
|  | Women | 176 (62.6) | 323 (61.6) |  |
| **Type of RD** | Osteomuscular | 20 (7.1) | 60 (11.4) | 0.138 |
|  | Nervous system | 92 (32.7) | 155 (29.5) |  |
|  | Congenital malformations | 40 (14.2) | 61 (11.6) |  |
|  | Ocular | 59 (20.9) | 95 (18.1) |  |
|  | Endocrine | 17 (6) | 49 (9.3) |  |
|  | Other | 53 (18.8) | 104 (19.8) |  |
| **Age at diagnosis** | | n (x̄) | n (x̄) | p value (Mann-Whitney U Test) |
|  |  | 277 (38.5) | 524 (37.1) | 0.053 |
