# Supplementary material for: Psychosocial impact at the time of a rare disease diagnosis
Source: PLoS One. 2023 Jul 28;18(7):e0288875. doi: 10.1371/journal.pone.0288875 (PMC10381039; doi:10.1371/journal.pone.0288875)
Supplement: S2 Table — (DOCX) [file pone.0288875.s002.docx]

**Supporting information**

**S2 Table**: Table showing the complete list of included RD in the study

| **Rare disease name** | **ORPHA code** |
| --- | --- |
| Acromegaly | 963 |
| Acute intermittent porphyria | 79276 |
| Addison disease | 85138 |
| Adult-onset cervical dystonia | 420492 |
| Alpha-1-antitrypsin deficiency | 60 |
| Amyotrophic lateral sclerosis | 803 |
| Antiphospholipid syndrome | 80 |
| Antisynthetase syndrome | 81 |
| Arachnoid cyst | 2356 |
| Arachnoiditis | 137817 |
| Arnold-Chiari malformation type I | 268882 |
| Autosomal dominant Charcot-Marie-Tooth disease type 2K | 99944 |
| Autosomal erythropoietic protoporphyria | 79278 |
| Autosomal recessive cerebelloparenchymal disorder type 3 | 1170 |
| Autosomal recessive spastic paraplegia type 76 | 488594 |
| Behçet disease | 117 |
| Benign concentric annular macular dystrophy | 251287 |
| Bullous pemphigoid | 703 |
| Calpain-3-related limb-girdle muscular dystrophy R1 | 267 |
| Caroli disease | 53035 |
| Catecholaminergic polymorphic ventricular tachycardia | 3286 |
| Chronic inflammatory demyelinating polyneuropathy | 2932 |
| Classic mycosis fungoides | 2584 |
| Clippers | 284448 |
| Combined dystonia | 98203 |
| Congenital fibrosis of extraocular muscles | 45358 |
| Congenital glaucoma | 98976 |
| Congenital pulmonary valvar stenosis | 3189 |
| Cowden syndrome | 201 |
| CREST syndrome | 90290 |
| Dermatomyositis | 221 |
| Disorder of fructose metabolism | 308463 |
| Distal myotilinopathy | 98911 |
| Dural sinus malformation | 97339 |
| Dystrophic epidermolysis bullosa | 303 |
| Ehlers-Danlos syndrome | 98249 |
| Eosinophilic granulomatosis with polyangiitis | 183 |
| Epidermolysis bullosa simplex | 304 |
| Epithelioid hemangioendothelioma | 157791 |
| Essential thrombocythemia | 3318 |
| Facioscapulohumeral dystrophy | 269 |
| Familial drusen | 75376 |
| Familial paroxysmal ataxia | 97 |
| Focal, segmental or multifocal dystonia | 1866 |
| Fragile X syndrome | 908 |
| Friedreich ataxia | 95 |
| Gaucher disease type 1 | 77259 |
| Glycogen storage disease due to muscle glycogen phosphorylase deficiency | 368 |
| Gyrate atrophy of choroid and retina | 414 |
| Hereditary angioedema type 1 | 100050 |
| Hereditary fructose intolerance | 469 |
| Hereditary spastic paraplegia | 685 |
| Hyperlipoproteinemia type 1 | 411 |
| Hypermobile Ehlers-Danlos syndrome | 285 |
| Idiopathic achalasia | 930 |
| Idiopathic hypersomnia | 33208 |
| Idiopathic intracranial hypertension | 238624 |
| Idiopathic multicentric Castleman disease | 570431 |
| Idiopathic pleuroparenchymal fibroelastosis | 494428 |
| Idiopathic pulmonary arterial hypertension | 275766 |
| Inclusion body myopathy with Paget disease of bone and frontotemporal dementia | 52430 |
| Indolent systemic mastocytosis | 98848 |
| Interstitial cistitis | 37202 |
| Isolated complex I deficiency | 2609 |
| Isolated cytochrome C oxidase deficiency | 254905 |
| Isolated microphthalmia-anophthalmia-coloboma | 2542 |
| Isolated optic neuritis | 499096 |
| Langerhans cell histiocytosis | 389 |
| Leber hereditary optic neuropathy | 104 |
| Legg-Calvé-Perthes disease | 2380 |
| Limb-girdle muscular dystrophy | 263 |
| Lymphangioleiomyomatosis | 538 |
| Lynch syndrome | 144 |
| Marfan syndrome | 558 |
| Melkersson-Rosenthal syndrome | 2483 |
| Mesial temporal lobe epilepsy with hippocampal sclerosis | 99701 |
| Mitochondrial myopathy | 206966 |
| Monoclonal mast cell activation syndrome | 529468 |
| Monosomy 9p | 261112 |
| Multiminicore myopathy | 598 |
| Multiple endocrine neoplasia type 2A | 247698 |
| Multiple endocrine neoplasia type 2B | 247709 |
| Myasthenia gravis | 589 |
| Narcolepsy | 619284 |
| Narcolepsy type 1 | 2073 |
| Neuralgic amyotrophy | 2901 |
| Neurofibromatosis type 1 | 636 |
| Neurofibromatosis type 2 | 637 |
| Non-acquired panhypopituitarism | 90695 |
| Oculopharyngeal muscular dystrophy | 270 |
| Oligodontia | 99798 |
| Osteochondritis dissecans | 2764 |
| Osteogenesis imperfecta | 666 |
| Perineural cyst | 65250 |
| Poland syndrome | 2911 |
| Polycythemia vera | 729 |
| Postpoliomyelitis syndrome | 2942 |
| Primary progressive aphasia | 95432 |
| Progressive cone dystrophy | 1871 |
| Progressive hemifacial atrophy | 1214 |
| Pseudoxanthoma elasticum | 758 |
| Pudendal neuralgia | 60039 |
| Pulmonary arterial hypertension associated with another disease | 275791 |
| Rare dystonia | 68363 |
| Rare pulmonary hypertension | 71198 |
| Renal nutcracker syndrome | 71273 |
| Retinitis pigmentosa | 791 |
| Sarcoidosis | 797 |
| Selective IgM deficiency | 331235 |
| Sjögren-Larsson syndrome | 816 |
| Spinocerebellar ataxia type 3 | 98757 |
| Stargardt disease | 827 |
| Steinert myotonic dystrophy | 273 |
| Sturge-Weber syndrome | 3205 |
| STXBP1-related encephalopathy | 599373 |
| Sympathetic ophthalmia | 79098 |
| Systemic capillary leak syndrome | 188 |
| Systemic mastocytosis | 2467 |
| Systemic sclerosis | 90291 |
| Thalidomide embryopathy | 3312 |
| Thomsen and Becker disease | 614 |
| Toxic oil syndrome | 227972 |
| Trigeminal neuralgia | 221091 |
| Tumor necrosis factor receptor 1 associated periodic syndrome | 32960 |
| Turner syndrome | 881 |
| Usher syndrome | 886 |
| Usher syndrome type 1 | 231169 |
| Usher syndrome type 2 | 231178 |
| Vascular Ehlers-Danlos syndrome | 286 |
| Vestibular schwannoma | 252175 |
| Von Willebrand disease type 1 | 166078 |
| X-linked adrenoleukodystrophy | 43 |
| X-linked Charcot-Marie-Tooth disease | 64747 |
